# Chemical profile and phytotoxic action of *Onopordum acanthium* essential oil

**DOI:** 10.1038/s41598-020-70463-7

**Published:** 2020-08-11

**Authors:** Caixia Wei, Shixing Zhou, Kai Shi, Chi Zhang, Hua Shao

**Affiliations:** 1 State Key Laboratory of Desert and Oasis Ecology, Xinjiang Institute of Ecology and Geography, Chinese Academy of Sciences, Urumqi, 830011 Xinjiang China; 2 Research Center for Ecology and Environment of Central Asia, Xinjiang Institute of Ecology and Geography, Chinese Academy of Sciences, Urumqi, 830011 China; 3 University of Chinese Academy of Sciences, Beijing, 100049 China; 4 Shandong Provincial Key Laboratory of Water and Soil Conservation and Environmental Protection, College of Resources and Environment, Linyi University, Linyi, 276000 China

**Keywords:** Biochemistry, Ecology

## Abstract

The potential of utilizing *Onopordum acanthium* essential oil and its major constituents as environment friendly herbicides was investigated. In total 29, 25, and 18 compounds were identified from flower, leaf, and stem oils, representing 94.77%, 80.02%, and 90.74% of the total oil, respectively. Flower and stem oils were found to be rich in *n*-alkanes, which accounted for 57.33% in flower oil, and 82.33% in stem oil. Flower oil exerted potent inhibitory activity on both receiver species, *Amaranthus retroflexus* and *Poa annua*, which nearly completely suppressed seed germination at 5 mg/mL, and β-eudesmol is the most likely responsible compound for its phytotoxicity; in comparison, leaf and stem oils exhibited much weaker inhibitory activity on *A. retroflexus*, and stimulatory effect on *P. annua* when tested concentration was below 2.5 mg/mL. Alkanes in the oils were found to exert relatively weak plant growth regulatory activity. This report is the first on the chemical profile and phytotoxic action of *O. acanthium* oil as well as the phytotoxicity of β-eudesmol.

## Introduction

The genus *Onopordum* (Asteraceae) comprises 50 species that distribute in continental Europe and central and southwest of Asia^1^. Among them, *O. acanthium* L.*,* also known as Scotch thistle, is a biennial species, and height of mature plants can reach up to 3 m. Flowering plants have white lanate stems that are ridged with spiny-margined wings; the plant bears purple flowers, the inflorescence is a panicle that bears capitula^2^. This plant is a competitive species against forage plants, and its flowering stalks form a barrier to livestock movement, which makes this plant an agricultural pest in the U.S., Canada, and other countries^2^. In China, *O. acanthium* can be found frequently as a dominant species distributing in the Tianshan mountains and the Junggar basin of Xinjiang province, which is known as the arid or semi-arid region located in northwest China^3–5^.

Worldwide, *O. acanthium* has been extensively used to treat cancers, inflammatory disorders, cardiovascular, and urogenital diseases^6–12,13^ For instance, Cardiodoron, a medicinal product made by *O. acanthium*, is well-tolerated for treatment of functional cardiovascular that shows positive effects in medical practice^11^. Phytochemical studies revealed that it might produce allelochemicals with phytotoxic activities, such as flavonoids and sesquiterpene lactones, which could attribute to its widespread over several continents^14^. Flower of *O. acanthium* is widely used as a coagulating agent in cheesemaking industry, and its young shoots as well as its first year roots are used in salads^15,16^. The oil obtained from *O. acanthium* can be used as a renewable fuel and chemical feedstock, and there are quite a few reports on the yield and chemical composition such as fatty acid composition, tocopherol, and mineral contents of its oil^17–20^.

Essential oils are a group of special natural substances known for biological activities such as antimicrobial, herbicidal, antioxidant, anti-inflammatory activities, and so on^21–25^. So far there is no report on the chemical constituents of *O. acanthium* essential oil. The objectives of the current study include: (i) determination of the chemical profile of the essential oils obtained from different plant organs of *O. acanthium*; (ii) evaluation of the phytotoxic activity of *O. acanthium* essential oils and the major components.

## Results

### Chemical constituents of *O. acanthium* essential oils extracted from different plant organs

Essential oils were extracted from fresh flowers, leaves, and stems of *O. acanthium* using hydrodistillation method. The phytochemical profiles of the essential oils obtained from different plant organs were analyzed via GC/MS, and the results were presented in Table 1. In total, 29, 25, and 18 compounds were identified from flower, leaf, and stem oils, representing 94.77%, 80.02%, and 90.74% of the total oil mass, respectively. All these oils were found to be rich in a type of particular compounds-*n*-alkanes (mainly C22–C29), such as tricosane, tetracosane, pentacosane, hexacosane, heptacosane, octacosane, nonacosane, etc., especially in flowers and stems, which accounted for 57.33% in the flower oil, and 82.33% in the stem oil. The major components of flower, leaf and stem oils differed significantly: pentacosane (12.41%), hexacosane (10.44%), β-eudesmol (10.10%), heptacosane (8.67%), tetracosane (8.43%), and tricosane (5.36%) were dominant in flower oil; leaf oil contain 1-Hexanol (14.88%), 3-Hexen-1-ol, (E)- (13.53%), 2-Hexen-1-ol, (E)- (12.18%), 2-Hexenal, (E)- (6.93%), nonanal (9.74%); on the other hand, stem oil was rich in nonacosane (16.44%), nonacosane, 2-methyl- (15.54%), hentriacontane (15.05%), and dotriacontane (13.73%).

**Table 1:** Chemical composition of the essential oils extracted from flowers, leaves and stems of *O. acanthium*.

Compounds	Number	Flower (%)	Leaf (%)	Stem (%)
2-Hexenal, (E)-	1	–	6.93	–
3-Hexen-1-ol, (E)-	2	–	13.53	1.17
2-Hexen-1-ol, (E)-	3	–	12.18	–
1-Hexanol	4	–	14.88	–
Heptanal	5	–	0.68	–
2-Amylfuran	6	–	0.73	–
(3E)-Hexenyl acetate	7	–	1.19	–
Nonanal	8	–	9.74	1.01
(+)-2-Bornanone	9	–	0.11	–
Methyl salicylate	10	–	0.16	–
3-p-Menthen-7-al	11	–	0.48	–
2-Decenal, (Z)-	12	–	0.33	–
Undecanal	13	0.28	0.71	–
Tridecane	14	–	1.00	0.10
2-Buten-1-one, 1-(2,6,6-trimethy…	15	–	–	0.16
β-Damascenone	16	0.89	1.70	–
β-Ionone	17	–	1.01	–
Phenol, 2,4-di-tert-butyl-	18	0.65	–	–
Pentadecane	19	–	–	0.73
Farnesan	20	–	0.61	–
Elemol	21	1.94	–	–
D-Nerolidol	22	1.53	–	–
Farnesene epoxide, E-	23	1.77	–	–
7-epi-γ-Eudesmol	24	1.17	–	–
β-Eudesmol	25	10.10	–	–
Bisabolol	26	0.65	–	–
Hexadecane	27	–	–	0.25
Laciniatafuranone F	28	–	0.36	–
cis-Davanone	29	–	5.91	–
1,2-Epoxyhexadecane	30	3.20	–	–
Heptadecane	31	–	–	4.71
Z-11-Hexadecenoic acid	32	1.38	–	–
9,12-Octadecadienal	33	2.30	–	–
Hexahydrofarnesyl acetone	34	2.17	–	–
Nonadecane	35	–	–	2.19
Dodecenyl succinic anhydride	36	0.09	–	–
Hexahydrofarnesyl acetone	37	1.59	–	–
2-Nonadecanone	38	0.12	–	–
Dibutyl phthalate	39	3.26	–	3.26
Phytol	40	2.06	–	–
Docosane	41	1.35	–	–
Tricosane	42	5.36	–	–
Octadecyl bromide	43	–	0.17	–
Phthalic acid	44	–	2.00	–
Tetracosane	45	8.43	–	3.31
Pentacosane	46	12.41	–	–
Hexacosane	47	10.44	0.44	0.09
Heptacosane	48	8.67	–	–
Octacosane	49	0.79	–	17.32
7-Hexyldocosane	50	4.45	–	–
1-Nonacosene	51	–	0.13	–
2-Methyloctacosane	52	–	4.34	0.22
Nonacosane	53	3.37	–	16.44
Triacontane	54	–	–	0.53
Nonacosane, 2-methyl-	55	–	–	15.54
Hentriacontane	56	2.06	–	15.05
Dotriacontane	57	–	–	13.73
Tetradecamethylhexasiloxane	58	–	0.70	–
Total		94.77	80.02	90.74

### Phytotoxic activity of flower, leaf and stem oils of *O. acanthium*

**Figure 1:**
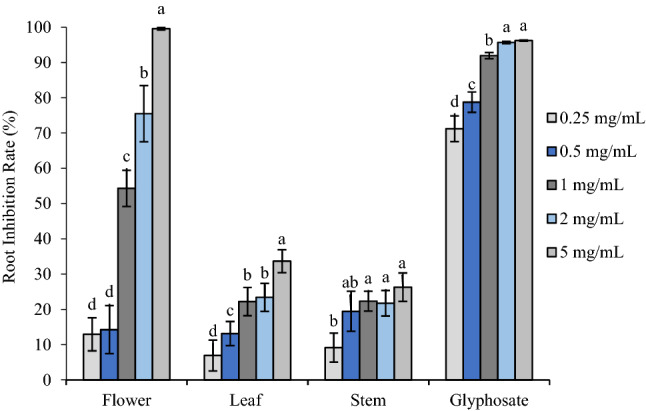
Phytotoxic effect of flower, leaf and stem oil of *O. acanthium* as well as glyphosate on radical elongation of *A. retroflexus* (*n* = 30). Each value is the mean of three replicates ± SE (*n* = 30). Significant difference (*p* < 0.05) was represented by different letters.

Flower, leaf, and stem oils (concentration tested ranged from 0.25–5 mg/mL) all exhibited significant plant growth regulatory activity against both receiver species (Figs. 1, 5). The oils posed inhibitory activity on root development of the dicot plant, *A. retroflexus*, with flower oil being the most potent oil, suppressing root elongation by 12.92% and 14.25% at 0.25 and 0.5 mg/mL. When the concentration of flower oil was increased to 1 mg/mL, the inhibitory effect was enhanced markedly, reaching 54.3% compared with the control. The suppressive effect was enhanced to 75.49% by 2.5 mg/mL oil, meanwhile 5 mg/mL flower oil almost completely killed the seedlings (99.60%).

**Figure 2:**
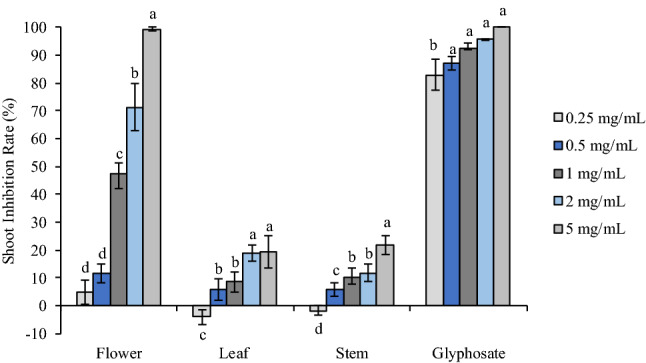
Phytotoxic effect of flower, leaf and stem oil of *O. acanthium* as well as glyphosate on shoot elongation of *A. retroflexus.* Each value is the mean of three replicates ± SE (*n* = 30). Significant difference (*p* < 0.05) was represented by different letters.

Although leaf and stem oils also exhibited negative impact on root growth of *A. retroflexus*, the strength was much weaker. Root length decreased to 6.89–33.66% of control when treated with leaf oil ranging from 0.25–5 mg/mL, and 9.15–26.28% with stem oil. It was obvious that strength of the oils’ phytotoxicity was much weaker compared with the commercial herbicide glyphosate, which reduced root length by 71.22%, 78.75%, 91.94%, 95.66% and 96.20% at 0.25, 0.5, 1, 2, and 5 mg/mL, respectively. Shoot development of *A. retroflexus* responded to the oils in a similar way, however both leaf and stem oils promoted shoot length by 3.91% and 5.91%, respectively (Figs. 2, 5).

**Figure 3:**
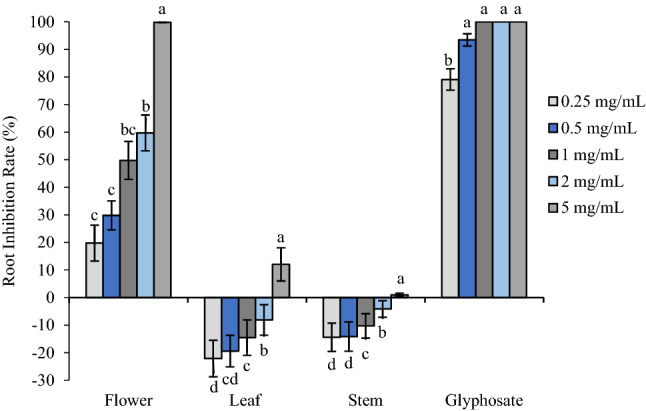
Phytotoxic effect of flower, leaf and stem oil of *O. acanthium* as well as glyphosate on radical elongation of *P. annua*. Each value is the mean of three replicates ± SE (*n* = 30). Significant difference (*p* < 0.05) was represented by different letters.

**Figure 4:**
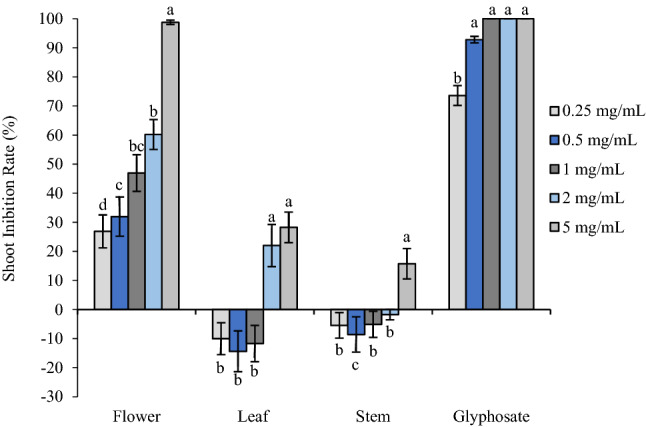
Phytotoxic effect of flower, leaf and stem oil of *O. acanthium* as well as glyphosate on shoot elongation of *P. annua*. Each value is the mean of three replicates ± SE (*n* = 30). Significant difference (*p* < 0.05) was represented by different letters.

**Figure 5:**
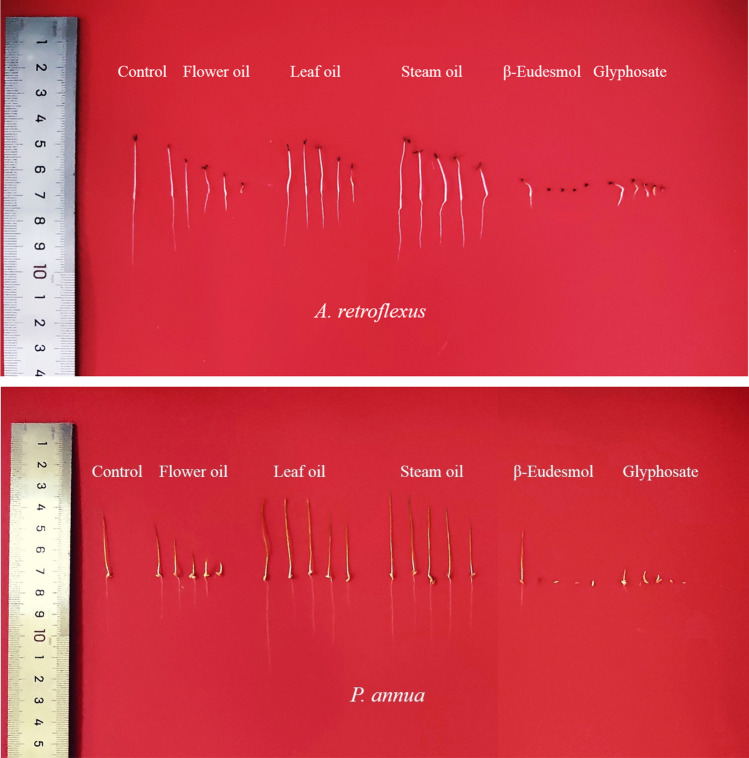
Phytotoxic effect of flower, leaf, and stem oil of *O. acanthium*, β-eudesmol, and glyphosate (concentration tested ranging from 0.25–5 mg/mL) on seedling growth of *A. retroflexus* and *P. annua*.

Flower oil exhibited similar inhibitory activity on *P. annua*, which was also much more sensitive to flower oil than leaf and stem oils. Root length was reduced to 49.75% and 59.72% of the control when treated with flower oil at 1 mg/mL and 2.5 mg/mL, respectively, and 5 mg/mL almost completely prohibited seed germination of *P. annua* (Figs. 3, 5). Similarly, glyphosate presented much stronger phytotoxic effect, inhibiting root elongation by 79.08% and 93.44% at 0.25 and 0.5 mg/mL, respectively, and starting from 1 mg/mL, seedling development was completely prohibited. On the other hand, leaf and stem oils promoted root development except the highest concentration tested (5 mg/mL), which reduced root length by 12.05% and 0.98%; leaf and stem oils stimulated root elongation by 8.13–22.11% and 4.14–14.41%, respectively. Like *A. retroflexus*, shoot growth of *P. annua* also displayed the same pattern as root growth (Figs. 4, 5).

### Phytotoxic activity of alkanes as major constituents of the essential oils

The essential oils produced by *O. acanthium* were rich in various alkane compounds, especially the flower and stem oils. Therefore, their possible involvement of the plant regulatory activity was assessed. In general, alkanes as the major constituents of the essential oils possessed relatively weak plant growth regulatory activity on receiver species. In terms of *A. retroflex*, alkanes stimulated root elongation by up to 26.97% (tetracosane at 0.25 mg/mL) when the applied concentration was between 0.25–0.5 mg/mL, and the strongest inhibitory effect (13.12%) was observed when the mixture of different alkanes were mixed at 5 mg/mL (Table 2). For *A. retroflex* shoot, only suppressive effect occurred, with pentacosane inhibiting shoot growth by 37.42% at 2.5 mg/mL (Table 3). As of *P. annua*, in general root development was inhibited, and the highest value was triggered by application of nonacosane at 5 mg/mL (Table 4); meanwhile, shoot length was promoted when alkane concentrations were lower than 2.5 mg/mL, and tricosane at 0.25 mg/mL enhanced shoot growth by 20.12% compared to the control. The mixture exhibited the most potent suppressive effect, resulting in 17.59% reduction on shoot elongation of *P. annua* (Table 5).

**Table 2:** Phytotoxic activity of alkanes on root growth of *A. retroflexus*.

	Concentration (mg/mL)	Tricosane	Tetracosane	Pentacosane	Hexacosane	Heptacosane	Octacosane	Nonacosane	Mixture
*A. retroflexus* Root inhibition (%)	0.25	− 10.22 ± 2.31^b^	− 26.97 ± 3.33^b^	− 9.99 ± 1.82^a^	− 16.38 ± 4.21^c^	− 12.10 ± 4.41^b^	− 9.29 ± 3.18^a^	− 14.09 ± 3.20^b^	− 15.26 ± 5.00^c^
	0.5	− 10.46 ± 4.41^b^	− 17.78 ± 4.13^ab^	− 4.01 ± 1.10^a^	− 13.70 ± 4.0^bc^	− 9.05 ± 2.30^b^	− 4.81 ± 1.24^a^	− 14.26 ± 3.23^b^	− 16.67 ± 9.33^c^
	1	− 2.38 ± 1.10^ab^	− 19.71 ± 4.70^ab^	0.76 ± 0.47^a^	− 9.78 ± 4.89^b^	− 1.91 ± 1.10^ab^	− 2.73 ± 1.55^a^	− 3.19 ± 3.92^a^	2.17 ± 4.29^b^
	2.5	− 3.31 ± 3.10^ab^	− 18.31 ± 3.65^ab^	0.90 ± 0.49^a^	− 1.32 ± 1.92^a^	− 2.38 ± 2.31^ab^	− 0.38 ± 0.34^a^	− 6.03 ± 3.25^a^	1.96 ± 1.93^b^
	5	8.05 ± 3.43^a^	− 9.06 ± 3.81^a^	2.29 ± 0.18^a^	− 0.38 ± 0.28^a^	7.67 ± 3.43^a^	− 0.77 ± 0.91^a^	1.26 ± 2.69^a^	13.12 ± 3.55^a^

^a^± Standard error (*n* = 30).

^b^Different letters represent a significant difference at *p* < 0.05 level according to Fisher’s LSD test.

**Table 3:** Phytotoxic activity of alkanes on shoot growth of *A. retroflexus*.

	Concentration (mg/mL)	Tricosane	Tetracosane	Pentacosane	Hexacosane	Heptacosane	Octacosane	Nonacosane	Mixture
*A. retroflexus* Shoot inhibition (%)	0.25	0.08 ± 0.55^b^	14.28 ± 2.62^b^	22.06 ± 3.49^b^	24.31 ± 1.93^a^	20.30 ± 4.06^b^	11.28 ± 2.85^b^	20.45 ± 2.97^a^	14.96 ± 2.79^a^
	0.5	0.07 ± 0.80^a^	14.79 ± 3.04^b^	24.55 ± 3.39^b^	23.06 ± 3.25^a^	28.21 ± 2.49^a^	17.54 ± 2.80^a^	19.55 ± 3.77^a^	17.29 ± 2.97^a^
	1	0.10 ± 0.40^a^	13.87 ± 1.92^b^	29.07 ± 2.41^ab^	25.78 ± 0.08^a^	28.32 ± 3.40^a^	19.30 ± 2.52^a^	22.81 ± 4.41^a^	16.79 ± 2.47^a^
	2.5	0.16 ± 0.89^a^	16.04 ± 1.91^b^	37.42 ± 4.54^a^	27.57 ± 13.77^a^	31.58 ± 3.27^a^	21.30 ± 2.46^a^	23.56 ± 4.96^a^	19.55 ± 3.10^a^
	5	0.14 ± 0.10^a^	30.00 ± 4.16^a^	36.84 ± 3.47^a^	29.74 ± 9.89^a^	30.52 ± 4.01^a^	21.70 ± 2.73^a^	23.78 ± 4.12^a^	19.23 ± 3.00^a^

^a^± Standard error (*n* = 30).

^b^Different letters represent a significant difference at *p* < 0.05 level according to Fisher’s LSD test.

**Table 4:** Phytotoxic activity of alkanes on root growth of *P. annua*.

	Concentration (mg/mL)	Tricosane	Tetracosane	Pentacosane	Hexacosane	Heptacosane	Octacosane	Nonacosane	Mixture
*P. annua* Root inhibition (%)	0.25	7.42 ± 3.79^b^	0.53 ± 0.47^b^	-8.13 ± 2.64^b^	− 0.68 ± 0.89^b^	16.54 ± 2.66^a^	0.06 ± 0.03^a^	8.66 ± 3.20^b^	7.29 ± 3.77^b^
	0.5	7.29 ± 3.28^b^	− 1.26 ± 1.13^b^	4.11 ± 2.52^a^	5.21 ± 3.11^b^	18.80 ± 2.73^a^	0.14 ± 0.02^a^	11.02 ± 2.59^ab^	11.83 ± 3.51^b^
	1	10.03 ± 3.55^ab^	4.41 ± 3.23^b^	3.39 ± 2.38^a^	14.40 ± 2.79^a^	20.30 ± 2.81^a^	0.13 ± 0.30^a^	12.79 ± 2.43^ab^	16.26 ± 3.84^b^
	2.5	13.98 ± 3.83^a^	16.26 ± 2.02^a^	6.46 ± 2.10^a^	16.03 ± 3.01^a^	21.80 ± 3.11^a^	0.18 ± 0.33^a^	19.30 ± 2.93^ab^	20.90 ± 2.75^ab^
	5	15.97 ± 2.96^a^	15.34 ± 2.39^a^	10.11 ± 3.02^a^	16.57 ± 2.76^a^	21.31 ± 2.68^a^	0.22 ± 0.04^a^	30.67 ± 2.30^a^	27.44 ± 2.37^a^

^a^± Standard error (*n* = 30).

^b^Different letters represent a significant difference at *p* < 0.05 level according to Fisher’s LSD test.

**Table 5:** Phytotoxic activity of alkanes on shoot growth of *P. annua*.

	Concentration (mg/mL)	Tricosane	Tetracosane	Pentacosane	Hexacosane	Heptacosane	Octacosane	Nonacosane	Mixture
*P. annua* Shoot inhibition (%)	0.25	− 20.12 ± 3.25^b^	− 6.23 ± 2.61^b^	− 16.93 ± 3.04^a^	2.99 ± 4.32^ab^	− 8.82 ± 2.46^b^	− 16.58 ± 3.43^b^	− 12.63 ± 3.31^b^	− 2.02 ± 1.36^c^
	0.5	− 19.21 ± 2.95^b^	− 4.21 ± 2.65^b^	− 13.16 ± 2.61^a^	2.62 ± 3.59^a^	− 9.68 ± 3.33^b^	− 12.22 ± 4.85^b^	− 10.26 ± 2.80^b^	− 9.47 ± 4.19^b^
	1	− 12.63 ± 2.88^b^	− 4.65 ± 4.11^b^	− 12.43 ± 2.82^a^	2.94 ± 3.84^a^	− 4.12 ± 2.98^ab^	− 3.90 ± 2.29^ab^	− 11.23 ± 3.65^b^	− 13.84 ± 3.15^b^
	2.5	− 7.98 ± 4.1^ab^	− 0.68 ± 3.70^ab^	− 13.14 ± 3.03^a^	3.13 ± 2.79^b^	− 1.14 ± 1.02^a^	− 4.47 ± 4.90^ab^	− 5.18 ± 3.23^ab^	− 11.85 ± 2.25^b^
	5	2.00 ± 3.41^a^	8.71 ± 2.98^a^	− 7.13 ± 4.77^a^	2.36 ± 3.24^ab^	2.46 ± 3.00^a^	7.63 ± 2.7^a^	5.26 ± 4.70^a^	17.59 ± 1.28^a^

^a^± Standard error (*n* = 30).

^b^Different letters represent a significant difference at *p* < 0.05 level according to Fisher’s LSD test.

### Phytotoxic activity of β-eudesmol

Phytotoxic assay demonstrated that the strength of flower oil was much higher than leaf and stem oils; further comparison revealed that flower oil was abundant in a unique compound, β-eudesmol. It is thus speculated that this compound might be responsible for the phytotoxic activity of the flower oil. To testify this hypothesis, the phytotoxic activity of β-eudesmol was further evaluated, and the results showed that indeed it possessed the same biological activity comparable to the flower oil (Tables 6, 7; Fig. 5). Starting from the lowest concentration, 0.25 mg/mL, over 50% reduction of root and shoot growth of both receiver plants were observed: root length was reduced by 79.46% and 63.34% for *A. retroflexus* and *P. annua*, and shoot length was suppressed by 60.37% and 68.96%, respectively. When the concentration was doubled to 0.5 mg/mL, seedling growth was nearly completely inhibited, and further increase in the dose resulted in 100% death of the seeds. The content of β-eudesmol was 10.16% in the total oil, therefore, if β-eudesmol was the only constituent responsible for the phytotoxicity of the oil, the phytotoxicity of 0.25 mg/mL β-eudesmol should be similar to 2.5 mg/mL oil, whilest 0.5 mg/mL β-eudesmol equals to the activity triggered by 5 mg/mL oil. In the assay, 0.25 mg/mL β-eudesmol caused 79.46% and 63.34% root reduction of *A. retroflexus* and *P. annua*, compared with 75.49% and 59.72% affected by flower oil at 2.5 mg/mL. Meanwhile, 0.5 mg/mL β-eudesmol caused 98.81% and 100% inhibition rate on root elongation of *A. retroflexus* and *P. annua*, compared with 99.6% and 100% on test species. In terms of shoot development, 0.25 mg/mL β-eudesmol caused 60.37% and 68.96% reduction of *A. retroflexus* and *P. annua*, compared with 71.31% and 60.20% of reduction under 2.5 mg/mL flower oil treatment; meanwhile, 0.5 mg/mL β-eudesmol reduced shoot elongation by 97.41% and 100%, compared with 99.43% and 98.76% triggered by 5 mg/mL flower oil. Thus, it was concluded that the single compound β-eudesmol was most likely the active responsible compound for the strong phytotoxicity of flower oil.

**Table 6:** Phytotoxic effect of β-eudesmol and glyphosate on seedling growth of *A. retroflexus*.

Concentration (mg/mL)	Root inhibition (%)		Shoot inhibition (%)	
	β-Eudesmol	Glyphosate	β-Eudesmol	Glyphosate
0.25	79.46 ± 3.25^b^	71.22 ± 0.35^d^	60.37 ± 6.21^b^	83.02 ± 0.75^e^
0.5	98.81 ± 0.68^a^	78.75 ± 0.46^c^	97.41 ± 1.57^a^	87.05 ± 0.25^d^
1	100 ± 0.00^a^	91.94 ± 0.19^b^	100 ± 0.00^a^	92.67 ± 0.20c
2.5	100 ± 0.00^a^	95.66 ± 0.13^a^	100 ± 0.00^a^	95.76 ± 0.18^c^
5	100 ± 0.00^a^	96.20 ± 0.13^a^	100 ± 0.00^a^	100 ± 0.00^a^

^a^± Standard error (*n* = 30).

^b^Different letters represent a significant difference at *p* < 0.05 level according to Fisher’s LSD test.

**Table 7:** Phytotoxic effect of β-eudesmol and glyphosate on seedling growth of *P. annua*.

Concentration (mg/mL)	Root inhibition (%)		Shoot inhibition (%)	
	β-Eudesmol	Glyphosate	β-Eudesmol	Glyphosate
0.25	63.34 ± 7.5^a^	79.08 ± 0.10^c^	68.96 ± 6.78^a^	73.60 ± 0.06^c^
0.5	100 ± 0.00^a^	93.44 ± 0.23^b^	100 ± 0.00^a^	92.80 ± 0.24^b^
1	100 ± 0.00^a^	100 ± 0.00^a^	100 ± 0.00^a^	100 ± 0.00^a^
2.5	100 ± 0.00^a^	100 ± 0.00^a^	100 ± 0.00^a^	100 ± 0.00^a^
5	100 ± 0.00^a^	100 ± 0.00^a^	100 ± 0.00^a^	100 ± 0.00^a^

^a^± Standard error (*n* = 30).

^b^Different letters represent a significant difference at *p* < 0.05 level according to Fisher’s LSD test.

## Discussion

Our study is the only report so far on the chemical composition and phytotoxicity of *O. acanthium* essential oil. Previously, the chemical composition of essential oils from another *Onopordum* species, *O. arenarium*, was investigated, and in total 29 and 25 compounds were identified from this plant’s flower and stem volatile oils, representing 91.6% and 89.2% of the oil mass, respectively. The oils were found to possess antioxidant activity, which were rich in long-chain hydrocarbons (23.3–36.4%), oxygenated long-chain hydrocarbons (31.5–33.8%), and oxygenated monoterpenes (14.4–6.6%), with palmitic acid being the most abundant constituent (25.5–28.7%)^26^. As of non-volatile compounds, phytochemical study revealed the presence of flavonoids such as apigenin, luteolin, scutellarein, coumarins such as aesculin and aesculetin, sesquiterpene lactones including derivatives of elemane, germacrane, eudesmane, and guaiane^1^. Watanabe et al. (2014) suspected that allelopathy might facilitate the invasion success of *O. acanthium*, therefore bioassay-guided isolation procedure was applied using wheat seeds as test species to investigate the chemicals in the leaves, which was extracted with different solvents; the CH_2_Cl_2_ extract showed the most potent phytotoxic activity, which was then further purified to yield 4 compounds, i.e. two flavonoids, pectolarigenin (1) and scutellarein 4′-methyl ether (2), and two sesquiterpene lactones, elemanolide 11(13)-dehydromelitensin b-hydroxyisobutyrate (3) and acanthiolide (4), among which they found compound 3 (IC_50_ = 1.794 × 10^–4^ M) strongly inhibited the growth of wheat coleoptiles and compound 1 (IC_50_ = 1.263 × 10^−3^ M) showed an intermediate effect^14^. To the best of knowledge, this is the first study on the chemical profile and phytotoxicity of this plant’s essential oil. Altogether 60 compounds from flower, leaf and stem oils of *O. acanthium* were identified, which were found to be rich in higher *n*-alkanes.

Alkanes are important industrial chemicals and energy resources that can be found rich in petroleum and natural gas. Although a number of plant species are found to produce essential oils containing alkanes as constituents, the amount is usually relatively low, except a single report on the chemical profile of *Asphodelus aestivus* flower essential oil, which was abundant in hexadecanoic acid (35.6%), pentacosane (17.4%), tricosane (13.4%), heptacosane (8.4%), heneicosane (4.5%), tetracosane (3.0%), and hexacosane (2.0). Polatoğlu et al. also identified nonacosane (16.18 ± 0.13%) and heptacosane (14.91 ± 0.17%) from the flower oil of *Arabis purpurea* Poech oil; it is interesting that similar to the current study, this oil was also obtained from the flowers of the donor plant^27^.

Besides, alkanes have been reported to be present in essential oils extracted from other Asteraceae plants. For instance, alkanes (C11-C27) accounted for 4.12% of the total mass of the essential oil of *Centaurea raphanina*, and 6.1% of *C. spruneri*^28^; other reports include that *Hemizonia fitchii* oil contained 1.7% alkanes (C25–C29)^29^, and 0.9% in *Sigesbeckia jorullensis* oil^30^. Compared with published studies, alkanes (C22–C29) including tricosane, tetracosane, pentacosane, hexacosane, heptacosane, octacosane, nonacosane accounted for 57.33% in flower oil, and 82.33% in stem oil, indicating the potential value of utilizing *O. acanthium* as a natural source to produce alkanes for industrial uses. It is noteworthy to mention that *O. acanthium* is a widespread species with high biomass that can adapt to various environmental conditions including arid and barren habitats, which makes it practical to be cultivated in large scale for industrial purpose.

This study indicated that flower oil possessed much stronger inhibitory effect compared with leaf and stem oils: the inhibition rate of flower oil on root growth of *A. retroflexus* was 2.96 and 3.79 times of leaf and stem oils, and on *P. annua* was 8.29 and 101.88 times of leaf and stem oils, respectively. Further experiment revealed that β-eudesmol (10.10%), which was a dominant compound in flower oil but not detected in leaf and stem oils, was most likely responsible for the potent phytotoxic activity of flower oil by comparing the strength of β-eudesmol and flower oil. β-eudesmol was found to possess antiangiogenic, antimutagenic, anticancer, antioxidant activity and so on, however there is no report on its phytotoxicity^21,31–36^.

β-Eudesmol has been previously reported to be abundant in *Drimys winteri* oil (7.27%), and in the drupe essential oil of *Citharexylum spinosum* as the most abundant component (33.1%) and other species; in some cases, essential oils containing β-eudesmol were reported to exert phytotoxic activity, such as *Geranium wilfordii* root oil (4.18% β-eudesmol), *Helichrysum italicum* essential oil (1.4% β-eudesmol), *Achillea wilhelmsii* essential oil (2.7% β-eudesmol), and *Rhynchosia minima* (0.79% β-eudesmol and 16.38% α-eudesmol, an analog of β-eudesmol), however it is difficult to ascribe the activity to certain constituent (s), especially considering the fact that in most cases β-eudesmol was not even the major components in the oils with phytotoxic activity. It is noteworthy to mention that the current report is the first on the phytotoxicity of β-eudesmol^37–39^.

Essential oils produced by various plant species are found to possess a number of biological activities including phytotoxic activity. As a matter of fact, not only essential oils but also their constituent such as thymol, β-myrcene, limonene, camphor, eucalyptol, geraniol, α- and β-pinene, borneol, etc. have been reported to play roles as plant growth regulatory agents^40–42^.

Besides, there are successful examples of utilizing essential oil or oil constituents as commercial herbicides. For example, the herbicide cinmethylin is the derivative of 1,4-cineole, which is a natural phytotoxin that can be found in the volatile oils of many plant species; clove oil is used as the major component in the commercial herbicide Burnout II (Bonide Products Inc., New York)^43,44^. The potent inhibitory activity of the flower oil and its major component, β-eudesmol, indicated their potential value of being further explored as environment friendly herbicide; meanwhile, the fact that the aboveground plant parts of *O. acanthium* can release volatile compounds into the surroundings support the hypothesis proposed by Watanabe that allelopathy might contribute to the widespread of this species^45,46^.

## Methods

### Plant materials

Aboveground plant parts of *O. acanthium* were harvested in Changji city, Xinjiang province, China, in July 2018 (N43°98′53″, E86°46′22″). Specimens were authenticated and identified by Dr. Wenjun Li, and a voucher specimen (XJBI018326) was deposited at the herbarium of Xinjiang Institute of Ecology and Geography, Chinese Academy of Sciences. Plant materials were separated into flowers, leaves, and stems for further process.

### Essential oil extraction

Essential oils were obtained by performing traditional boiling hydrodistillation procedure. Fresh flowers, leaves, and stems of *O. acanthium* (200 g were used for each plant organ) were laid out in the flask containing water and the unit is carried to boiling for 4 h. The mixture of water–oil was produced in the flask then condensed in the condenser, where the oils were harvested. The same procedure was repeated until enough oils were obtained for GC/MS analysis and the phytotoxic activity assay. The essential oils were dried over anhydrous Na_2_SO_4_ and stored at 4 °C for the following GC/MS analysis and bioassay experiments.

### GC/MS analysis

GC/MS was performed to determine the chemical constituents of *O. acanthium* oil using a 7890A/5975C GC–MS system with an automatic injector (Agilent Technologies, Palo Alto, CA, USA) equipped with FID and a DB-5MS 5% Phenyl Methyl Silox column (30 m × 0.25 mm; film thickness 0.25 μm). The experimental conditions were programmed as follows: Helium (carrier gas) at a flow rate of 1 mL/min; the temperature of the oven was first maintained at 60 °C for 5 min, then programmed from 60 to 280 °C at a rate of 3 °C/min; injector and detector temperature: 280 °C; sample injection volume: 0.1 µL (the sample was first diluted in hexane at high-performance liquid chromatography grade at 1:100, v/v); split ratio: 50:1; mass spectra were taken at 70 eV with mass range from m/z 40–800 amu. Identification of essential oil constituents was achieved based on comparison of their mass spectra and retention indices (RI, calculated by linear interpolation relative to retention times of a standard mixture of C7–C40 *n*-alkanes at the same chromatographic conditions s) with the National Institute of Standards and Technology (NIST 14) library database and a home-made library obtained from genuine compounds, as well as those given in the published literature^11,25^. Relative amounts of individual constituents were calculated based on GC peak areas without using correction factors.

### Phytotoxic bioassay

Strength of phytotoxicity of *O. acanthium* essential oils obtained from different plant parts along with the oil’s major components were investigated by performing bioassays against *Amaranthus retroflexus* L. (dicot) and *Poa annua* L. (monocot) using glyphosate (Roundup, Monsanto Co., St. Louis, MO, USA) being the positive control; these 2 species were chosen because they can be found growing alongside *O. acanthium* in the same habitat. The essential oils’ major constituents including tricosane, tetracosane, pentacosane, hexacosane, heptacosane, octacosane, nonacosane, and β-eudesmol were purchased from Alfa Aesar^®^ (Shanghai, China); purity of all compounds were 98%. Seeds of receiver species were surface sterilized with 2% sodium hypochlorite before use. The oils and the major components were first dissolved in DMSO (0.1% final concentration) and then diluted with distilled water containing Tween 80 (final concentration 0.02%) to give solutions at 0.25, 0.5, 1, 2.5, and 5 mg/mL for the assay (a preliminary experiment was conducted and results demonstrated that DMSO and Tween 80 at such concentrations did not affect seedling growth of *A. retroflexus* and *P. annua* significantly). Each petri dish (φ 5 cm) received 2 mL solutions (controls received 2 mL of distilled H_2_O containing 0.1% DMSO and 0.02% Tween 80) and 10 sterilized seeds of receiver species. Petri dishes were sealed with parafilm and kept in a growth cabinet at 25 °C with a 12-h photoperiod. *A. retroflexus* and *P. annua* seedlings were measured after 5 days and 7 days of incubation, respectively, due to relative slow development of *P. annua* seedlings*.* Three replicates were performed for the assays (*n* = 30)^46^.

### Statistical analyses

ANOVA (*p* < 0.05) was applied to determine the significance of phytotoxicity triggered by the *O. acanthium* essential oils and the major components, followed by analysis using Fisher’s LSD test (*p* < 0.05). Experimental results were statistically analyzed using SPSS 13.0 software package and presented as means of 3 replicates ± standard error.

## Conclusion

The current study is the first report on the chemical profile and phytotoxicity of *O. acanthium* essential oil, and on the phytotoxicity of β-eudesmol, which is speculated to be the responsible compound of flower oil, indicating their potential of being further explored as bioherbicides; flower oil and stem oil were found to be rich in *n*-alkanes, implying that *O. acanthium* has the potential to be further explored as a natural source to produce alkanes for industrial purpose.
